# Traitement préventif intermittent à la sulfadoxine-pyriméthamine chez la femme enceinte et effet sur le poids de naissance du bébé: application de la politique à 3 doses en zone urbaine au Sud Bénin en 2017

**DOI:** 10.11604/pamj.2019.34.155.19357

**Published:** 2019-11-20

**Authors:** Chabi Olaniran Alphonse Biaou, Alphonse Kpozehouen, Yolaine Glèlè-Ahanhanzo, Gloria Ayivi-Vinz, Abdou-Rahim Ouro-Koura, Colette Azandjèmé

**Affiliations:** 1 Département d'Epidémiologie et de Biostatistiques, Institut Régional de Santé Publique, Université d'Abomey-Calavi, Bénin; 2 Centre de Formation en Santé Publique, Lomé, Togo; 3 Département de Promotion de la Santé, Institut Régional de Santé Publique, Université d'Abomey-Calavi, Bénin

**Keywords:** Paludisme, grossesse, petit poids de naissance, prévention et contrôle, Benin, Malaria, pregnancy, low birth weight, prevention and control, Benin

## Abstract

**Introduction:**

Le paludisme est un problème de santé publique majeur, responsable de nombreuses complications durant la grossesse parmi lesquelles le retard de croissance intra utérin et les hypotrophies. L'objectif de ce travail était de déterminer l'effet du respect des 3 doses du traitement préventif intermittent (TPI) à la sulfadoxine-pyrimétamine (SP) sur le poids de naissance des nouveau-nés de la zone sanitaire Cotonou II et III.

**Méthodes:**

Il s'agit d'une étude transversale qui a porté sur 630 femmes en période post partum résidant dans la zone sanitaire Cotonou II-III et sélectionnées par une technique de sondage à deux degrés. Les données ont été recueillies par un questionnaire et une fiche de dépouillement. Pour l'analyse, les données ont été pondérées et nous avons utilisé l'analyse de variance pour la comparaison des moyennes et une comparaison de proportions avec le test de chi² assorti d'une estimation de la force de l'association par l'odds ratio (OR) et son intervalle de confiance à 95%.

**Résultats:**

Le respect des 3 doses du TPI à la SP était de 34,08% (IC95%: [24,84% - 43,30%]). On note un gain significatif de poids de naissance de 264,5g (p < 0,001) chez les mères qui avaient reçu plus de 3 doses de SP et la probabilité d'avoir un bébé ayant un faible poids de naissance était plus faible (OR = 0,45; p = 0,001) chez ces mères comparativement à celles qui avaient reçu moins de 3 doses de sulfadoxine-pyriméthamine.

**Conclusion:**

Cette étude révèle une faible observance de la nouvelle politique à 3 doses du traitement préventif intermittent à la sulfadoxine-pyriméthamine dans la zone sanitaire de Cotonou II et III, mais elle témoigne de son potentiel de contribution à la réduction du risque de faible poids de naissance. Des stratégies doivent donc être mises en œuvre pour renforcer son application en vue de la prévention du paludisme et de ses conséquences pour les cibles vulnérables.

## Abstract (en)

**Introduction:**

Malaria is a major public health problem, responsible for many complications during pregnancy including inadequate intra-uterine growth and hypotrophies. The aim of this study was to determine the adherence to the 3 doses of sulfadoxine-pyrimethamine-based intermittent preventive treatment (IPT) on birthweight in the Cotonou health zone II and III.

**Methods:**

We conducted a cross-sectional study focusing on 630 women in the postpartum period residing in the Cotonou health zone II and III and selected using a two stage sample. Data were collected through a questionnaire and a data processing form. For analysis, data were weighted and we used variance analysis to compare means and to compare proportions with Chi-square Test including estimates regarding the strength of the association by odds ratio (OR) and confidence interval at 95%.

**Results:**

Adherence to the 3 doses of sulfadoxine-pyrimethamine-based intermittent preventive treatment was 34.08% (CI95%: 24.84% 43.30%]. A significant birthweight-gain of 264.5 g (p < 0.001) in mothers who had received more than 3 doses of sulfadoxine-pyrimethamine-based intermittent preventive treatment and chances of having low birth weight baby was lower (OR = 0.45; p = 0.001) among these mothers compared to those who had received less than 3 doses of sulfadoxine pyrimethamine.

**Conclusion:**

This study highlights low adherence to the new 3-dose regimen of sulfadoxine-pyrimethamine-based intermittent preventive treatment in the Cotonou health zone II and III, but it reflects its potential to contribute to the reduction of the risk of low birth weight. Strategies must therefore be implemented to strengthen its application for prevention of malaria and of its consequences on vulnerable targets.

## Introduction

Le paludisme est un problème majeur de santé publique mondial. L'Organisation Mondiale de la Santé (OMS) estime à 216 millions, le nombre de cas enregistrés dans le monde en 2016. La région africaine de l'OMS continue de supporter une part élevée de la charge mondiale avec 90% des cas de paludisme et 91% des décès imputables au paludisme en 2016 [1]. Les enfants âgés de moins de cinq ans et les femmes enceintes constituent les couches les plus vulnérables. En effet, le paludisme chez la femme enceinte se caractérise par une anémie maternelle pouvant conduire au décès maternel ou à une mort in utero; le retard de croissance intra utérine, la prématurité et le faible poids de naissance (FPN) sont également des conséquences fréquentes [1-4]. Il est prouvé que dans les régions endémiques du paludisme, le risque de FPN attribuable au paludisme varie de 8 à 14% chez les nouveau-nés à terme et de 8 à 36% chez les prématurés. Le paludisme maternel serait responsable de plus de 35% des causes évitables de FPN, facteur de risque de mortalité infantile le plus important [5, 6]. Pour réduire le fardeau lié au paludisme sur grossesse, l'OMS recommande un paquet d'intervention tripartite composé de la distribution et l'utilisation des moustiquaires imprégnées d'insecticide, la prise en charge efficace des cas et une utilisation de la Sulfadoxine Pyrimétamine (SP) pour le Traitement Préventif Intermittent (TPI) du paludisme dans les zones de transmission modérée à sévère [1, 7]. La SP est le seul médicament actuellement recommandé pour la prévention du paludisme chez la femme enceinte et il convient de souligner qu'elle continue d'être bénéfique à la mère et à son bébé, même dans les régions de résistance de la SP en traitement curatif du paludisme [1]. Il est désormais recommandé un schéma d'au moins 3 doses de TPI à base de SP dès le second trimestre de la grossesse, à partir de la seizième semaine d'aménorrhée lors de chaque consultation prénatale (CPN) programmée jusqu'à l'accouchement. Chaque dose doit être administrée à au moins un mois d'intervalle [1, 8]. Dans la zone sanitaire (ZS) urbaine de Cotonou II et III, comme dans les autres ZS du Bénin, le TPI à la SP a été introduit en 2006 avec des recommandations d'administration d'au moins 2 doses de SP avant l'accouchement. Les nouvelles recommandations de passage à un minimum de 3 doses ont été introduites en 2016 au niveau national. La présente étude est destinée à contribuer à l'amélioration des stratégies de prévention du paludisme chez les femmes enceintes à travers la production de l'évidence scientifique des effets positifs des nouvelles recommandations. Elle vise à documenter le respect des 3 doses du TPI à la SP conformément aux nouvelles recommandations, et son effet sur le poids de naissance des nouveau-nés dans un contexte urbain.

## Méthodes

**Cadre de l'étude:** l'étude a été menée dans la ZS Cotonou II et III qui est l'une des quatre ZS du département du Littoral (Sud Bénin). Il s'agit d'une zone sanitaire strictement urbaine qui regroupe les quatre premiers arrondissements de la ville de Cotonou (Capitale économique du Bénin). Elle est limitée au Sud par l'Océan Atlantique, au Nord par le lac Nokoué, à l'Est par la commune de Sèmè- Kpodji et à l'Ouest par la lagune de Cotonou. Elle couvre une superficie de 25 km^2^.

**Type d'étude:** il s'est agi d'une étude transversale, qui s'était déroulée de mai à juillet 2017.

**Échantillonnage:** elle a porté sur les femmes en période post partum (PP) résidant dans la zone sanitaire Cotonou II-III depuis au moins six mois, ayant accouché au cours des douze derniers mois (entre juin 2016 et mai 2017), et disposant d'un carnet de CPN pour le dernier accouchement. Était exclue de l'étude toute femme répondant aux critères d'inclusion, et sous traitement par le cotrimoxazole ou qui aurait refusé d'être interviewée ou de terminer l'entretien. Les femmes ont été sélectionnées selon une méthode probabiliste avec la technique de sondage à deux degrés. La base de sondage était constituée de la liste des quartiers/villages de la ZS Cotonou II-III. La taille de l'échantillon a été calculée sur la base de la couverture en TPI 2 (46,4%) dans la zone sanitaire Cotonou II-III en 2015 [9] suivant la formule de Schwartz avec une précision de 5% et un effet de sondage k = 1,5. La taille de l'échantillon était: n = 573. Cette dernière a été majorée de 10% pour tenir compte des non-réponses. Le nombre de femmes à enquêter était donc de 630 accouchées. À partir de la liste des quartiers et villages de la ZS et du nombre de naissances attendues, 30 quartiers ont été sélectionnées par échantillonnage aléatoire systématique. Pour la sélection des concessions, dans chaque quartier tiré, l'enquêteur s'était placé au centre du quartier/village en tirant au sort une direction par la méthode de la bouteille. Dans cette direction, il a commencé par la deuxième concession à droite puis a évolué toutes les trois concessions. Dans chaque concession, les femmes en PP répondant aux critères d'inclusion étaient identifiées et recensées puis deux parmi elles étaient tirées au hasard pour l'enquête. Toute concession visitée ne comportant pas nos cibles était remplacée par la concession suivante et ainsi de suite jusqu'à l'obtention de l'effectif attendu par village ou quartier.

**Technique, outils de collecte et variables:** les données ont été collectées par entretien avec les mères éligibles et par exploitation de leur carnet de consultation de la dernière grossesse au moyen d'un questionnaire et d'une fiche de dépouillement. Les deux outils ont fait l'objet d'un pré-test. La variable d'intérêt était le poids de naissance. Les autres variables étaient constituées du nombre de doses de SP reçues par la mère durant sa dernière grossesse ainsi que des caractéristiques des mères (âge, parité, gestité, nombre de CPN) et le sexe du nouveau-né.

**Traitement et analyse des données:** après la collecte des données, les fiches ont été dépouillées manuellement afin de vérifier la complétude des données recueillies ainsi que leur cohérence. Les données ont ensuite été saisies à l'aide du logiciel Epi Data 3.1 et analysées avec le logiciel Stata 14. Toutes les analyses ont été pondérées. Les proportions ont été calculées pour les variables qualitatives. Les moyennes ainsi que leur écart-type ainsi que la médiane et son intervalle interquartile (IIQ) ont été déterminées selon le cas pour les variables quantitatives. Ont donc été considérées dans cette analyse, les mères pour lesquelles l'information du poids de naissance du bébé était disponible dans le carnet. Nous avons utilisé l'analyse de variance pour la comparaison des moyennes et une comparaison de proportions avec le test de chi^2^ assorti d'une estimation de la force de l'association par l'odds ratio (OR) et son intervalle de confiance à 95%. Le seuil de significativité était de 5%.

**Considérations éthiques et déontologiques:** les répondantes ont été informées par les enquêteurs des objectifs de l'enquête, de son caractère anonyme ainsi que du libre choix de participer ou non. Le questionnaire ne leur a été administré qu'après obtention de leur consentement verbal éclairé. Notons que nous avons obtenu l'autorisation des autorités administratives de la zone sanitaire de la ZS Cotonou II-III avant toute collecte de données.

## Résultats

Au total, 567 mères ont été considérées dans cette étude.

**Les caractéristiques de la population étudiée:** l'âge médian des mères était de 27 ans (IIQ: 24-32) avec une prédominance de la tranche d'âge allant de 25 à 29 ans (33,07%). Il s'agissait principalement des femmes paucigestes (54,57%) et paucipares (54,60%). Elles avaient en moyenne assisté à 4,69 ± 1,83 CPN au cours de leur dernière grossesse; 72,68% d'entre elles avaient assisté à au moins quatre CPN. Notons que 50,84% des nouveau-nés étaient de sexe masculin, soit un sex-ratio de 1,01 (Tableau 1).

**Tableau 1:** Répartition selon les caractéristiques des mères et des nouveau-nés enquêtés dans la zone sanitaire Cotonou II et II, mai 2017 (n = 567)

Variables	Modalités	Effectifs	Fréquence (%)
**Age de la mère (ans)**	< 20	27	4,83
	20 - 24	141	23,45
	25 - 29	184	33,07
	30 - 34	146	26,67
	≥ 35	69	11,98
**Gestité**	1	125	20,87
	2-3	299	54,57
	≥ 4	143	24,56
**Parité**	1	154	25,92
	2-3	300	54,60
	≥ 4	113	19,48
**Nombre de CPN**	< 4	152	27,32
	≥ 4	415	72,68
**Sexe du nouveau-né**	Masculin	286	50,84
	Féminin	281	49,16

**Figure 1:**
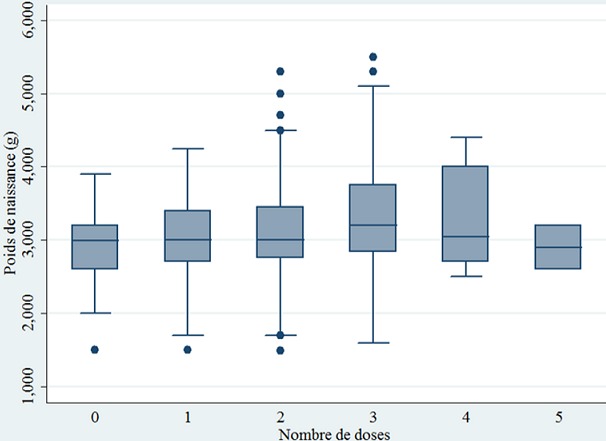
Répartition du poids moyen de naissance selon le nombre de dose de TPI-SP reçu, zone sanitaire Cotonou II et II, Mai 2017 (n = 567)

**Figure 2:**
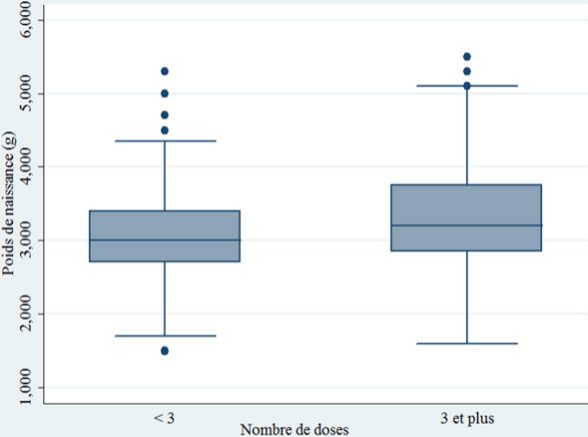
Répartition du poids moyen de naissance selon le respect de la nouvelle politique à 3 doses de SP, zone sanitaire Cotonou II et II, Mai 2017 (n = 567)

**Nombre de doses de SP et effet sur le poids de naissance:** parmi les 567 mères, 194 soit 35,42% avaient reçu au moins trois doses de TPI à la SP durant leur dernière grossesse. Le poids moyen de naissance du bébé était de 3138,91 ± 621,23 g. Il évolue significativement (p < 0,001) selon le nombre de doses de SP reçu par la mère durant son dernier accouchement (Figure 1). En effet, entre 0 et 3 doses, on constate une augmentation du poids moyen du bébé avec un effet dose réponse. Ainsi le poids moyen de naissances passe avec une évolution ascendante de 2891 g ± 438g à zéro dose de SP à 3313 g ± 689 g à 3 doses de TPI-SP, soit un gain de 422 g. On ne note plus de progression positive au-delà de 3 doses. Quand on tient compte de la répartition du poids moyen de naissance selon le nombre recommandé de doses de SP, on constate que le poids moyen de naissance des bébés dont les mères avaient reçu au moins trois doses de TPI-SP (3036,42 g ± 567,78 g) était significativement supérieur (p < 0,001) à ceux dont les mères avaient reçu moins de trois doses de SP (3300,96g ± 681g); soit un gain de poids moyen de 264,5g (p < 0,001) (Figure 2). Quand on s'intéresse au faible poids de naissance, 55 cas, soit 10,90% (IC95%: [6,50% - 15,30%]) ont été enregistrés et il est constaté que le nombre de doses du TPI à la SP était significativement (p < 0,001) associé au FPN. En effet, la probabilité de survenue du FPN était 2,22 fois plus élevée (p = 0,001) chez les mères qui ont reçu moins de 3 doses de SP par rapport à celles qui avaient reçu au moins 3 doses de SP.

## Discussion

**Synthèse des principaux résultats:** la proportion de femmes qui avaient reçues au moins trois doses de TPI à la SP durant leur dernière grossesse était de 35,42% dans la zone sanitaire Cotonou II et III en 2017. Le poids moyen de naissance du bébé était de 3138,91 ± 621,23 g et il évolue significativement selon le nombre de doses de SP reçu par la mère durant son dernier accouchement. Pour ce qui concerne le faible poids de naissance, il a été constaté chez 1 enfant sur 10. La probabilité de survenue du FPN était plus élevée chez les mères qui ont reçu moins de 3 doses de SP par rapport à celles qui avaient reçu au moins 3 doses de SP.

**Forces et limites de l'étude:** notre technique d'enquête a permis d'avoir un échantillon qui était représentatif de la population des gestantes de la zone sanitaire Cotonou II et III. En ce qui concerne les limites de cette étude, des biais éventuels ne sont pas à occulter et sont à prendre en compte dans l'interprétation des résultats. Le biais de sélection pourrait être lié à la probabilité de non-réponse ou de refus de participation à l'enquête. Pour contrôler ces refus et garantir une taille minimale pour notre échantillon, nous avions prévu un taux de non-réponse de 10% lors du calcul de la taille de l'échantillon. Il pourrait également être lié aux critères d'inclusion, car nous n'avons pris que les femmes qui avaient encore leur carnet. Le choix de cette option en vue d'être très spécifique améliore la qualité des informations recueillies car l'objectivité du carnet est plus fiable que la déclaration de l'enquêtée. Toutefois, on pourrait envisager qu'il y a plus de femmes « observantes » dans ce groupe de femmes qui conservent leur carnet car elles sont peut-être plus conscientes de son importance. En ce qui concerne les biais d'informations, le fait que nous n'ayons recruté que des accouchées pourrait en être une source. C'est la raison pour laquelle la période post partum a été restreinte à un an après l'accouchement afin de limiter les biais de mémoire. Par ailleurs aucune conclusion causale ne peut être tirée des résultats de cette étude en raison de la nature instantanée des études transversales.

**Discussion des principales différences:** l'objectif visé par cette étude était de documenter l'effet du TPI à la SP sur le poids de naissance des nouveau-nés dans une zone sanitaire urbaine au Sud du Bénin. L'étude révèle que les trois doses de TPI-SP recommandées par l'OMS ont été reçues par 35,42% de femmes soit une femme sur trois, tandis que 71,96% des femmes ont observé au moins deux doses. Cela traduit un faible niveau d'observance de cette nouvelle politique dans cette zone urbaine du Bénin. Notons que la plupart des pays endémiques et concernés par la nouvelle recommandation de l'OMS ont changé de politique sur le nombre de doses de TPI à appliquer aux femmes enceintes très récemment. Au Bénin, elle a effectivement démarré en 2016. Et si la zone sanitaire Cotonou II et III est passée d'une observance de 46,4% en 2015 à 71,96% en seulement deux ans, on peut s'attendre à une amélioration significative de la nouvelle politique dans les années à venir [9]. Ce mérite peut être attribué d'une part à l'effort déployé par Programme National de Lutte contre le Paludisme du Bénin pour l'amélioration de la couverture en TPI. Il pourrait également être attribué au profil urbain de notre cadre d'étude. En effet comme toute capitale économique, la commune de Cotonou jouit d'une densité populationnelle importante et est exposée à une multitude de services de santé publics et privés [10]. Cet atout pourrait expliquer le fait que dans la présente étude, environ sept femmes enceintes sur dix utilisaient réellement les services de CPN (au moins 4 CPN) tel que le recommande l'OMS [11]. Cette situation est tout à fait favorable à l'utilisation adéquate du TPI dans la mesure où ce sont lors de ces séances que l'administration de la SP se fait. Le niveau de respect de la nouvelle politique au TPI retrouvé dans cette étude était proche de ceux rapportés par Azizi *et al.* ainsi que Diarra *et al.* respectivement au Malawi: 29,8% [12] et au Mali: 36,7% [13]. Cela pourrait s'expliquer par le fait que ces pays ont démarré la mise en œuvre de la nouvelle politique de l'OMS dans les mêmes périodes. Au Ghana où la politique actuelle recommande depuis 2014 au moins cinq doses de SP, Owusu-Boateng et Anto ont rapporté en 2017 un respect du TPI à 3 doses de SP de 87,5% [14]. Pour ce qui concerne l'effet du TPI sur le poids de naissance, nous avons noté un gain significatif de poids de naissance en faveur des nouveaux nés de mères ayant observé les trois doses du TPI. Ce constat reste superposable aux données de la littérature [1, 2, 15, 16]. Par ailleurs, les résultats révèlent que le risque de FPN était plus élevé chez les mères qui avaient reçu moins de 3 doses à l'image de ce que rapporte la littérature sur la question [17]. Dans un contexte où la résistance du P. Falciparum à la SP se fait de plus en plus constater pour le traitement curatif dans plusieurs pays endémiques dont le Bénin [18], la prévention du paludisme gestationnel et de ses conséquences par l'utilisation de la SP continue de faire ses preuves. Malgré les diverses réticences, la SP demeure à ce jour, la seule molécule recommandée par l'OMS pour la prévention du paludisme gestationnel [19]. Les politiques existantes doivent alors maintenir le cap pour un contrôle effectif du paludisme chez cette cible considérée comme étant une priorité en matière de lutte contre le paludisme. Il est clair qu'une politique à 3 doses de TPI à la SP est plus contributive qu'une politique à 2 doses, cependant, les résultats de la présente étude ne confirment pas la contribution d'une politique à 4 ou à 5 doses de SP par rapport à une politique à 3 doses en ce qui concerne le poids de naissance du bébé. Sur cet indicateur, la politique à cinq doses proposée par certains pays reste donc tout à fait discutable, mais il est tout à fait plausible qu'une politique à 4 ou à 5 doses de SP contribue à la résolution d'autres problèmes que celui du faible poids de naissance du bébé. Cependant, il est important de nuancer les résultats de la présente étude sur cette question vue que l'effectif des femmes ayant reçu 4 et 5 doses de TPI à la SP est faible et ne peut être suffisant pour tirer des conclusions définitives; d'où la nécessité de mener des études complémentaires pour mieux clarifier ce point d'ombre.

## Conclusion

Le respect de la politique à trois doses de SP recommandée pour la prévention du paludisme gestationnel est faible dans la zone sanitaire Cotonou II et III; néanmoins son effet bénéfique sur le poids de naissance du bébé est clairement démontré. Il est donc d'autant plus opportun de poursuivre avec cette politique. Pour cela, il faut identifier les goulots d'étranglement ainsi que les stratégies pertinentes et contextualisées afin de contribuer à la mise en œuvre de cette politique de prévention du paludisme.

### Etat des connaissances actuelles sur le sujet

- Le traitement préventif intermittent à la sulfadoxine-pyriméthamine est l'une des recommandations de l'OMS pour la prévention du paludisme chez la femme enceinte;
- Les recommandations actuellement en vigueur dans les pays sont passées de l'ancienne politique à deux doses de Sulfadoxine-Pyriméthamine à une nouvelle politique avec un minimum de 3 doses de Sulfadoxine-Pyriméthamine.

### Contribution de notre étude à la connaissance

- Le niveau de respect de la politique à trois doses de traitement préventif intermittent à la sulfadoxine-pyriméthamine dans la zone sanitaire Cotonou II et III est faible;
- Le poids moyen de naissance du bébé évolue de manière ascendante selon le nombre de doses de SP reçues par la mère durant son dernier accouchement, mais il ne semble pas avoir d'amélioration au-delà de 3 doses;
- La probabilité de survenue du faible poids de naissance est moindre chez les mères qui ont respecté la politique à trois doses durant le dernier accouchement.

## Conflits d’intérêts

Les auteurs ne déclarent aucun conflit d’intérêts.
